# Computational Prediction of miRNA Genes from Small RNA Sequencing Data

**DOI:** 10.3389/fbioe.2015.00007

**Published:** 2015-01-26

**Authors:** Wenjing Kang, Marc R. Friedländer

**Affiliations:** ^1^Science for Life Laboratory, Department of Molecular Biosciences, The Wenner-Gren Institute, Stockholm University, Stockholm, Sweden

**Keywords:** miRNA, microRNA, gene prediction, next-generation sequencing data

## Abstract

Next-generation sequencing now for the first time allows researchers to gage the depth and variation of entire transcriptomes. However, now as rare transcripts can be detected that are present in cells at single copies, more advanced computational tools are needed to accurately annotate and profile them. microRNAs (miRNAs) are 22 nucleotide small RNAs (sRNAs) that post-transcriptionally reduce the output of protein coding genes. They have established roles in numerous biological processes, including cancers and other diseases. During miRNA biogenesis, the sRNAs are sequentially cleaved from precursor molecules that have a characteristic hairpin RNA structure. The vast majority of new miRNA genes that are discovered are mined from small RNA sequencing (sRNA-seq), which can detect more than a billion RNAs in a single run. However, given that many of the detected RNAs are degradation products from all types of transcripts, the accurate identification of miRNAs remain a non-trivial computational problem. Here, we review the tools available to predict animal miRNAs from sRNA sequencing data. We present tools for generalist and specialist use cases, including prediction from massively pooled data or in species without reference genome. We also present wet-lab methods used to validate predicted miRNAs, and approaches to computationally benchmark prediction accuracy. For each tool, we reference validation experiments and benchmarking efforts. Last, we discuss the future of the field.

## miRNA Biology

microRNAs (miRNAs) are a class of small RNAs (sRNAs) around 22 nucleotides in length. They are never translated, but post-transcriptionally reduce the output of protein coding genes (Kloosterman and Plasterk, 2006; Bushati and Cohen, 2007; Farazi et al., 2008; Ghildiyal and Zamore, 2009). They have been found in all animals studied, in numbers that appear to correlate with organismal complexity, for instance, nematodes have around 200 miRNA genes while humans have more than 3000 (Kozomara and Griffiths-Jones, 2011; Friedländer et al., 2014). Mutant animals that are void of miRNAs either die at early embryonic stages or have severe developmental defects, showing the importance of the regulation they infer (Bernstein et al., 2003; Giraldez et al., 2005; Morita et al., 2007; Wang et al., 2007). More than half of all protein coding transcripts are estimated to be under regulation of miRNAs in one or more cellular contexts (Friedman et al., 2009). Thus, it is not surprising that miRNAs are involved in numerous biological contexts, ranging from formation of cell identify to development (Stefani and Slack, 2008).

### miRNA biogenesis

**Figure 1:**
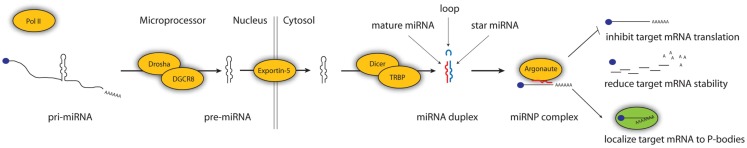
**miRNA biogenesis and function**. miRNAs are transcribed as primary transcripts or are sometimes derived from exons or introns of hosts transcripts. Characteristic hairpin RNA structures are recognized by Drosha and DGCR8 and cleaved out. The hairpin is exported to the cytosol and cleaved by Dicer, which is a part of the canonical RNA interference pathway, releasing three products: the two miRNA strands (the “mature” or “guide” strand and the “star” or “passenger” strand) and the terminal loop. The guide strand is then bound by an Argonaute protein, which is part of the miRNP effector complex. Once thus bound, the miRNA can bind to target sites, often located in the 3′ UTR of protein coding transcripts, and guide the effector complex to inhibit translation of the target, cause its degradation, or relocate it to subcellular foci, where they are no longer accessible to the translation machinery.

The majority of miRNAs are transcribed by Polymerase II and have features similar to protein coding transcripts: a 5′ cap, exons, and a poly(A)-tail (Figure 1). Each of the primary transcripts harbors one or more characteristic RNA hairpin structures around 60 nucleotides in length. While in the nucleus, these structures can be recognized by the Microprocessor complex, consisting of Drosha and DGCR8 proteins, which cleave the hairpin out of the primary transcript (Denli et al., 2004; Gregory et al., 2004; Han et al., 2004; Landthaler et al., 2004). The hairpin is then exported to the cytosol, where it undergoes a second cleavage by Dicer, a canonical component of the RNA interference pathway (Bernstein et al., 2001; Hutvagner et al., 2001; Ketting et al., 2001; Knight and Bass, 2001). The cleavage releases three products: the mature miRNA guide strand, the miRNA passenger strand, and the loop. These three products fall in determined positions: the guide and the passenger form an RNA duplex with two nucleotides 3′ overhangs, while the loop consists of the terminal end of the hairpin, positioned between the guide and the passenger strands (Ha and Kim, 2014). While the loop and the passenger strands are generally degraded as bi-products of the biogenesis, the guide miRNA remains bound to an Argonaute protein, which is part of the miRNP complex. It is not always the same strand that is fated to be bound to the Argonaute protein, in the case of many miRNA hairpins either strand can be incorporated and repress targets (Okamura et al., 2008; Guo and Lu, 2010; Yang et al., 2011). The mature miRNA can guide the effector complex to target sites, typically located in 3′ UTRs of mRNAs, through partial base complementarity (Lai, 2002; Bartel, 2009). Once bound, the complex reduces protein output of the transcript, either by destabilizing it through shortening of the poly-A tail, inhibiting its translation or by re-localizing it to subcellular ribo-protein particles, where it is inaccessible to the translation machinery (Filipowicz et al., 2008; Huntzinger and Izaurralde, 2011). Some miRNAs follow non-canonical biogenesis pathways, but are believed to function like the canonical sequences (Ha and Kim, 2014). Altogether, it is estimated that around 60% of all human protein coding transcripts are regulated by miRNAs in one or more cellular conditions (Friedman et al., 2009).

### miRNAs in human disease

Given the prevalence of miRNA regulation, it is not surprising that miRNAs have been involved in numerous human diseases. These regulators appear to play particularly critical roles in cancers, where they can function as onco-genes or tumor suppressors. For instance, the miR-17–92 cluster is found to be up-regulated in several cancers (He et al., 2005), and miR-15 and miR-16 are often deleted in leukemias (Cimmino et al., 2005). Although some miRNAs can function as onco-genes, they are in most cases down-regulated individually or collectively in cancers (Medina and Slack, 2008). miRNAs are important in cell differentiation and formation of cell identity, and often cancer cells revert to more undifferentiated states. In addition to cancers, miRNAs have been involved in many types of diseases including: cardiovascular, immunological, neurodegenerative, and psychiatric (Taft et al., 2010; Esteller, 2011). In disease, miRNA function can be perturbed in several ways: by down-regulation of the biogenesis factors (Hill et al., 2009), by mutation in the miRNA locus (Mencia et al., 2009), by loss or gains of the miRNA genes (Zhang et al., 2006b), or by epigenetic changes such as hypermethylation (Davalos et al., 2012). There are also cases where disease is caused by mutations that destroy (Christensen et al., 2009) or create (Abelson et al., 2005) target sites in the 3′ UTR of protein coding transcripts.

Before the role of a miRNA in a given disease can be investigated, it must be discovered and annotated. Many miRNAs have specific expression patterns and may not be highly expressed outside the particular tissue that is studied, and may not yet have been discovered. Therefore, miRNA prediction is an important first analysis step of sRNA-seq analysis in clinical context. miRNA prediction can also be used for basic research, when annotating the complement of regulatory RNAs in emerging model systems. The purpose of this review is to present the methods used to discover new animal or human miRNA genes from sRNA-seq data. We will focus on published methods that can be downloaded and run, without the user needing to implement algorithms as software by him/herself. We will discuss the strengths of the distinct methods, and will reference the studies in which the methods have been benchmarked computationally. Thus, this review can serve as a platform for the reader to decide which method is ideally suited for his miRNA prediction use case. Finally, we will present low and high-throughput methods to validate the discovered miRNA candidates.

## miRNA Prediction

### Prediction from genome sequence

The biogenesis of miRNAs is key to their discovery. When the field was still young and little data were available, researchers would search the genome sequences for loci that would give rise to RNA hairpin structure if transcribed. These methods have combined structure prediction with either scoring (Lai et al., 2003; Lim et al., 2003; Ohler et al., 2004; Wang et al., 2005) or rules-based (Dezulian et al., 2006; Zhang et al., 2006a) or machine-learning classification (Nam et al., 2005; Jiang et al., 2007; Sheng et al., 2007) of the hairpin features. Some of the methods have incorporated conservation information into the prediction; in fact, one approach has used phylogenetic shadowing to detect the characteristic conservation profile of miRNAs, where the miRNA strands are more conserved in sequence than the terminal loop (Berezikov et al., 2005). However, it is impossible to know from the genome DNA sequence if a locus is really transcribed and gives rise to mature miRNAs. Thus, considering the size of most animal genomes, these methods yield many false positive hairpins that are either not transcribed or do not interact with the biogenesis factors. For instance, in the human genome, around 11 million loci would give rise to hairpin structures if transcribed (Bentwich, 2005), but only a few thousands of them are actually cleaved to mature miRNAs (Kozomara and Griffiths-Jones, 2011; Friedländer et al., 2014).

### Sanger sequencing

For an unbiased detection of miRNAs, methods were developed to directly sequence sRNAs. This was done by separating them from other transcripts on high-resolution gels, and sequencing by Sanger sequencing (Lagos-Quintana et al., 2001; Lau et al., 2001; Lee and Ambros, 2001). Because of the limited throughput of this technology, typically just a few hundreds of sRNAs were detected, and many of these would be degradation products of longer transcripts such as mRNAs, rRNAs, and tRNAs, or even from un-annotated transcripts. To ensure that the predicted miRNAs were genuine, researchers would filter out sequences mapping to known non-miRNA transcript annotations, and would require that the predicted miRNA was located in a loci that could give rise to a hairpin transcript (Ambros et al., 2003). More specifically and in accordance with miRNA biogenesis, the predicted sequence should be located on a hairpin arm. Further, if two sequences should locate to the same hairpin, it was required that they should form a duplex with two nucleotide 3′ overhangs, as expected from Dicer processing.

### Next-generation sequencing

In 2006, the first next-generation sequencing instruments became commercially available, allowing orders of magnitude increase in data generation. For instance, the current Illumina HiSeq 2500 instruments can sequence around one billion sRNAs in <2 days. This sequencing power can be distributed between several experiments, but still sRNA-seq studies detect millions of transcripts per sample. Since a mammalian cell typically contains on the order of 100,000 miRNA transcripts (Calabrese et al., 2007), this means that sequences that are present in less than one molecule per cell can still be detected. This also holds for other clades, for instance, the *lsy-6* miRNA, which is expressed in only a single neuron in the entire nematode body (Johnston and Hobert, 2003), is now routinely detected in sRNA-seq experiments (unpublished results).

The sensitivity of these sequencing methods means that very lowly expressed sRNAs other than miRNAs are also detected. These can include short interfering RNAs (siRNAs) and piwi-interacting RNAs (piRNAs) but can also be rare degradation products of longer transcripts like rRNAs, tRNAs, and mRNAs or un-annotated transcripts. In addition to this, there is now emerging evidence that transcripts like tRNAs can undergo endonucleolytic cleavage at specific positions to produce functional sRNAs (Chen and Heard, 2013). Altogether, this means that sRNAs sequenced in a single experiment can originate from millions of distinct loci in the human genome (Friedländer et al., 2008). The methods that were developed to predict miRNAs from Sanger sequencing should only handle a few thousand loci. Therefore, they are not specific enough to be applied to next-generation sequencing data, and produce numerous false positives. These false positives are transcribed and form hairpins, but the sRNAs generated from them are degradation products resulting from normal RNA turnover. Thus, accurately identifying the miRNAs in this complex landscape of sRNAs is a daunting task.

To reduce false positives, methods to predict miRNAs from sRNA-seq employ post-filtering steps beyond what is used for Sanger sequencing. The next-generation discovery methods almost all require the presence of a hairpin structure, and the formation of a duplex if both miRNA strands are detected. In addition, many methods require that the candidate precursors do not overlap known non-miRNA annotations (Berninger et al., 2008). Hairpins that pass these requirements are then exposed to a further filter step. These steps can be rule-based or can involve probabilistic scoring or machine learning (see below). The features that are evaluated can be divided into *structure* features and *signature* features (Friedländer et al., 2008). The first reflect how well the hairpin structure conforms to known miRNA precursors. For instance, most of the nucleotides in the putative duplex should be base paired, and the hairpin should not contain large bulges besides the terminal loop. Some methods also require that the structure should be energetically stable, as this is a hallmark of genuine miRNA hairpins. The *signature* is a measure of how well the distribution of sequenced RNAs fit in the hairpin structure. For instance, every sequenced RNA should correspond to either guide or passenger strand, or to the terminal loop. The guide and passenger RNAs should form a duplex with two nucleotide 3′ overhangs, as is typical of Dicer processing. Further, it is expected that the candidate miRNA guide strand is detected several times, given the sensitivity of next-generation sequencing. Last, since it is known that processing of Drosha and Dicer produces clearly defined 5′ ends, the sequenced RNAs should align neatly in this end (Ruby et al., 2006).

Besides the core prediction methods, source for predicting miRNAs differ in other respects. This includes the mapping tool, whether read pre-processing is provided, whether the tool has a graphic user interface or must be operated on the command line and whether additional analyses like expression analyses and target predictions are supported. Also, some methods are not just applicable for animal miRNAs, but also for plant sequences. Finally, some methods have been tested by computational benchmarking in several studies and their predictions validated in the wet-lab. In the following section, we describe the tools of the field in alphabetical order (Table 1).

**Table 1:** **Tools for predicting animal miRNAs from sRNA-seq data**.

Tool	Algorithm	Mapping tool	Tested in plants	Performance comparison	Validated in wet-lab	Pre-process data	Quantifies expression	Target prediction	User interface
**GENERAL TOOLS**									
deepBlockAlign	Read block alignment	Not included	Yes	Langenberger et al. (2012), and Pundhir and Gorodkin (2013)	No	No	No	No	Graphics, webserver
miRanalyzer	Random forest	Prefix tree	No	Hackenberg et al. (2009)	See below	Partial	Differential expression	MiRanda and TargetScan	Graphics, webserver
miRanalyzer (update)	Random forest	Bowtie	Yes	An et al. (2013), Friedländer et al. (2012), Hackenberg et al. (2011) Hansen et al. (2014), Pundhir and Gorodkin (2013), and Williamson et al. (2013)	RT-PCR (Smith et al., 2013), Northern blot (Mayoral et al., 2014)	Yes	Differential expression	TargetSpy	Graphics, webserver, and standalone
miRCat	Rules-based	PatMaN	Yes	Moxon et al. (2008)	RT-PCR (Kohli et al., 2014, and Pandey et al., 2014), Northern blot (Donaszi-Ivanov et al., 2013)	Yes	Yes (mirprof), differential expression (colide)	PAREsnip	Graphics, webserver, and standalone
miRDeep	Bayesian	Megablast	No	An et al. (2013), Friedländer et al. (2008, 2012), Hendrix et al. (2010), and Williamson et al. (2013)	Northern blot (Friedländer et al., 2008, 2009), RT-PCR (Friedländer et al., 2012)	No	Yes	No	No graphics, standalone
miRDeep2	Bayesian	Bowtie	No	An et al. (2013), Friedländer et al. (2012), Hansen et al. (2014), and Williamson et al. (2013)	Knock-down (Friedländer et al., 2012), RT-PCR (Metpally et al., 2013)	Yes	Yes	No	Graphics, standalone
miRDeep*	Bayesian	Bowtie (java version)	No	An et al. (2013), and Hansen et al. (2014)	RT-PCR, knock-down (An et al., 2013)	Yes	Yes	TargetScan	Graphics, standalone (java software)
MIReNA	Rules-based	Megablast	Yes	An et al. (2013), Friedländer et al. (2012), and Mathelier and Carbone (2010)	Knock-down (Friedländer et al., 2012)	No	No	No	No graphics
miREvo	Bayesian	Bowtie	No	No	No	Yes	Yes	No	Graphics, standalone
miRExpress	Sequence homology	Custom mapping tools	No	No	No	Yes	Yes	No	No graphics, standalone
miRTRAP	Rules-based	Not included	No	An et al. (2013), Friedländer et al. (2012), and Hendrix et al. (2010)	Knock-down (Friedländer et al., 2012), Northern blot (Hendrix et al., 2010)	No	No	No	No graphics
**MASSIVELY POOLED DATA**									
miRdentify	Feature scoring	Bowtie	No	Hansen et al., 2014	RT-PCR (Hansen et al., 2014)	Yes	No	No	No graphics
**PREDICTION WITHOUT REFERENCE GENOME**									
MirPlex	Support vector machine	Not included	Yes	Mapleson et al. (2013)	Knock-out (Mapleson et al., 2013)	No	No	No	No graphics
MIRPIPE	Sequence homology	BLASTN	No	Kuenne et al. (2014)	No	Yes	Yes	No	Graphics, webserver, and standalone

*Algorithm: the core algorithm for identifying miRNAs. Mapping tool: software used to trace sequenced RNAs to the reference sequences. Tested in plants: if the method has been benchmarked with plant data. Performance comparison: studies that have benchmarked the performance of the tool. Validated in wet-lab: studies that have validated predicted miRNA candidates with experimental methods. Given the overall number of miRNA studies, this list may not be exhaustive. Pre-process data: tools that prepare the FASTQ sequence data for the mapping and prediction steps. Quantifies expression: tools that report estimated miRNA abundances. In addition, some tools report miRNAs that are differentially expressed between samples. Target prediction: tools that predict targets of candidate miRNAs. User interface: tools that have a graphic user interface (as opposed to being operated from the command line). Tools that are run on a webserver (as opposed to being installed and run on a local machine)*.

## Specific Algorithms

### deepBlockAlign

deepBlockAlign is innovative in that it provides advanced scoring of the read signature, but does not evaluate the RNA structure (Langenberger et al., 2012; Pundhir and Gorodkin, 2013). deepBlockAlign uses a variant of Needleman–Wunsch to identify blocks of mapped reads that have similar features, including read begin positions and block height. In a second step, similar groups of blocks are identified using a variant of the Sankoff algorithm. These groups of blocks correspond to gene loci. To predict novel miRNAs, the method finds loci that have block features similar to known miRNAs. While the profiles might be different for plants and animals, or specific to particular tissues or pathological conditions, the method can compare to all known profiles from the entire miRBase database of miRNAs, giving it good coverage. Since this method does not evaluate the RNA structure, it can predict miRNAs that do not have canonical structure, or whose conformation is not easily predicted by computational methods. Alternatively, it can be combined with down-stream structure analysis, to further improve specificity^1^.

### miRanalyzer

miRanalyzer first removes reads that map to known miRNAs or other transcripts (Hackenberg et al., 2009). The remaining reads are considered as potential new miRNAs. They are evaluated as miRNAs using a random forest machine learning approach. The classifier is initially trained on a set of known miRNAs from human, rat, or nematode and dozens of features are considered, including energetics, structure, bulges, and the number of reads mapping. The tool has fitted parameters for each species analyzed and on publication provided packages for seven commonly used species. miRanalyzer is available through a webserver, making it easily accessible for biologists with little computational experience^2^.

### miRanalyzer (update)

miRanalyzer (update) is an improved version with several new features. It uses bowtie (Langmead et al., 2009) for much faster and less memory-intensive mapping, and it includes parameter packages for 31 species, including 6 plants (Hackenberg et al., 2011). In addition, it can perform differential expression analysis of the profiled miRNAs and can predict targets using the TargetSpy tool. In addition to the web server version, it has a stand-alone version that can be downloaded and run on local machines. miRanalyzer predictions have been validated with several wet-lab methods (Smith et al., 2013; Mayoral et al., 2014). Since miRanalyzer often predicts more new miRNAs than do other tools, it is well suited for studies where the predictions will be filtered by additional computational tools or by high-throughput wet-lab validations^2^.

### miRCat

miRCat has been used successfully to predict miRNAs in several plants (Szittya et al., 2008; Pantaleo et al., 2010; Mohorianu et al., 2011) and has recently been adapted to animal sequences, including butterflies (Surridge et al., 2011). miRCat uses a rules-based approach that eliminates candidates with features that are not consistent with miRNA biogenesis (Moxon et al., 2008; Stocks et al., 2012). Numerous features are investigated, including the number of read stacks in the locus, the number of reads mapping anti-sense to the locus, the size of bulges in the candidate miRNA duplex, the number/fraction of paired nucleotides in the duplex and in the hairpin, and the energetic stability of the hairpin. miRCat is part of a suite, the UEA workbench, which includes numerous computational tools, some which can be applied to the analysis of non-miRNA small RNA sequences. miRCat predictions have been validated in several systems (Donaszi-Ivanov et al., 2013; Kohli et al., 2014; Pandey et al., 2014). Since it was developed for plant miRNAs that are more variable in structure, it could be well suited for detecting animal miRNA hairpins that are not typical for this clade^3^.

### miRDeep

miRDeep first filters all candidates whose structure and read signature are inconsistent with Drosha/Dicer processing (Friedländer et al., 2008). In the next step, the fit of the structure and signature to an explicit model of miRNA biogenesis is scored using Bayesian statistics. Specifically, miRDeep scores the number of reads supporting biogenesis, the presence of a miRNA passenger strand, the presence of a conserved miRNA seed and the absolute and relative energetic stability of the hairpin. While miRDeep can be run on data filtered for known non-miRNA annotations, it can perform robust prediction without this filtering. This means that miRNAs derived from non-canonical host transcripts, such as snoRNAs, can be identified (Ender et al., 2008). Further, it does not require parameters fitted to specific species, meaning that it is not at a disadvantage when mining emerging model systems. The tool has been extensively benchmarked and validated by experimental methods (Friedländer et al., 2008, 2009; Metpally et al., 2013), and has been adapted by several other research groups (Yang and Li, 2011; Yang and Qu, 2012; Wu et al., 2013)^4^.

### miRDeep2

miRDeep2 improves the previous version, primarily by making more robust predictions when faced with very deep sequencing data (Mackowiak, 2011; Friedländer et al., 2012). This includes improved excision of candidate hairpins from the genome, allowing for anti-sense miRNAs and moRs (see miRTRAP below). In addition, the tool has been improved in terms of computational efficiency, implementing better tools like bowtie (Langmead et al., 2009), and it features graphics output. Last, it has been tested in seven species, using the exact same parameters, and introduces knock-down of key proteins necessary for miRNA maturation to validate that novel candidates depend on the miRNA biogenesis pathways for their expression^4^.

### miRDeep*

miRDeep* is an extension of the first miRDeep algorithm, and incorporates many improvements similar to miRDeep2, although it was developed by a separate research group (An et al., 2013). It features pre-processing, bowtie mapping, improved precursor excision, and target prediction for known and novel miRNAs. The tool has an extensive graphical user interface and is implemented entirely in java without requiring any pre-dependent computational tools, making it portable and easy to install. The computational efficiency makes it run on a home computer^5^.

### MIReNA

MIReNA is a flexible tool to predict novel miRNAs from known miRNA sequences, next-generation sequencing data, long transcripts, or hairpin precursors (Mathelier and Carbone, 2010). It uses a rules-based scheme with sharp cut-offs to classify miRNAs based on five criteria: the lack of base pairing in the mature miRNA, the difference in length between the two candidate miRNA strands, the fraction of base-paired nucleotides in the hairpin, and two measures of energetic stability. As a second filtering step, it considers only hairpins where the sequenced RNAs map in consistence with Drosha/Dicer processing. MIReNA can consider several potential miRNA duplexes within one precursor structure, e.g., within multiple stem precursors, giving it the potential to predict non-canonical miRNAs^6^.

### miREvo

miREvo build on the miRDeep2 predictor (above) but extends it for evolutionary analyses (Wen et al., 2012). Specifically, it uses whole-genome alignments to identify miRNA homologs in related species. It also includes tools to compare expression of miRNA homologs across species, if sRNA-seq data are available for both species. It uses modified prediction parameters for plant analyses^7^.

### miRExpress

miRExpress is a tool for profiling miRNA expression from sRNA-seq data (Wang et al., 2009). However, it includes a function to predict miRNAs based on sequence homology. It maps each read that does not correspond to a known reference miRNA against miRBase sequences, keeping only perfect matches. These reads are then mapped against the reference genome, and the structure evaluated with the mfold structure prediction software (Zuker, 2003)^8^.

### miRTRAP

miRTRAP uses a rules-based approach with two filtering steps (Hendrix et al., 2010). In the first one, all candidate miRNAs whose structure and read signatures do not conform to Drosha/Dicer processing are eliminated. In the second step, all candidates that are not located in sRNA deserts are removed. This second step builds on the observation that miRNAs typically generates blocks of sRNAs with few or no sequenced RNA mapping to the anti-sense strand or in the general vicinity. In addition to this innovative filtering step, miRTRAP has high accuracy when predicting miRNAs with moRs, which are sRNAs generated from the flanks of the precursor hairpin. This development was necessary, as the tool was initially developed for identifying miRNAs in sea squirt, a species unusually rich in moRs (Shi et al., 2009)^9^.

## Special Applications

### Massively pooled data

Many researchers who apply miRNA prediction tools to sequencing data want to mine their own in-house data. These could be sequences from an emerging model organism, or from a human tissue of interest. The tools described above are all optimized for analyzing a limited number of data sets, ranging from maybe 1 to 20 sets. However, some studies compile all the available sRNA-seq data for a given species to give the best possible miRNA annotation. There are numerous advantages to pooling tens or hundreds of datasets (Friedländer et al., 2014). First, if the guide and passenger strands are detected in two distinct data sets, combining the information can allow analysis of the duplex features. Second, lowly expressed miRNAs might not be well profiled in single datasets, where it is difficult to evaluate the read signature. Third, since sRNA-seq library preparation involves a PCR amplification step, there is no guarantee that 10 sequencing reads in 1 dataset do not correspond to a single over-amplified sRNA. In contrast, if the same sequence is detected in data from 10 distinct tissues, this provides independent evidence that the biogenesis is common. Massively pooled sRNA-seq data have previously been used to predict miRNAs in general (Friedländer et al., 2014), or of the specific mirtron class (Ladewig et al., 2012). These are hairpins, which are released by intronic splicing rather than Drosha cleavage. Some mirtrons are short and their hairpin ends are defined by the splice signals, while others are longer, and one end is trimmed to define the hairpin end (Berezikov et al., 2007; Okamura et al., 2007; Ruby et al., 2007). In addition, the miRBase database employs massively pooled data to refine the miRNA annotations and define a high-confidence set of sequences (Kozomara and Griffiths-Jones, 2014). The software used in these studies has, however, not been published, so the methods are not described in detail here.

#### miRdentify

miRdentify has recently been released to the public to analyze massive pooled data (Hansen et al., 2014). It requires that both guide and passenger miRNA strands are detected and evaluates 10 features of the structure and signature, including precision of 5′ end processing, two nucleotide 3′ overhangs, and several aspect of stability. For each feature, the cut-off is set so that 1% of known miRNAs is excluded. Together, the requirement for detection of both strands and the 10 features constitute stringent criteria that produce miRNA candidates with features similar to known hairpins (Hansen et al., 2014). The method thus, to some extent, trades off sensitivity to report high-quality candidates^10^.

### Prediction without a reference genome

The majority of miRNA prediction tools require a reference genome as input to enable the excision of miRNA hairpin sequences, whose RNA structures and signatures are considered as key features for miRNA prediction. However, even though the price of next-generation sequencing technologies decreases, only a handful of model species have fully assembled high-quality reference genomes. Thus, many researchers rely on emerging model species without reference genomes, and novel methods are needed to discover new miRNAs in order to further study their function. One way to address this problem is to use a closely related species genome as proxy reference sequence to identify conserved miRNA. Such a study has been undertaken to discover mosquito miRNAs by mapping the sRNA-seq against the genomes of three related insect species (Etebari and Asgari, 2014). For this purpose, the miRanalyzer tool was used, and it was found that the prediction accuracy is affected by the evolutionary distance between the species of interest and the proxy species. Overall, the most abundant and conserved miRNAs were identified in this study, but the approach might be less successful for species that do not have closely related species with genome sequences.

#### MirPlex

MirPlex is a tool that requires only sRNA datasets as input with no genome sequences needed (Mapleson et al., 2013). It uses a multi-stage process to identify genuine miRNA duplexes. First, all overlapping sequences are assembled into contigs, and contigs that are too long to be miRNAs are discarded (> 30 nucleotides). Second, the remaining sequences are copied into two duplicate datasets followed with separate filter pathways to obtain candidate miRNA guide and miRNA passenger sequences. Last, the candidate miRNA guide and miRNA passenger sequences are then paired into duplexes for the classification. The core algorithm of MirPlex uses a support vector machine to classify genuine miRNA duplexes based on 20 features that divided into three categories: the size of sequences in the duplex, the stability of the duplex, and the nucleotide composition of the duplex. However, MirPlex depends on the presence of both strands in a miRNA duplex for prediction, and so cannot discover miRNAs unless the less abundant passenger strand is also detected by the sequencing^11^.

#### MIRPIPE

MIRPIPE identifies miRNAs through sequence homology (Kuenne et al., 2014). It collapses duplicate reads and removes those that have only been sequenced few times. It then further collapses sequences that only differ in the 3′ end and last maps the remaining sequences against known miRBase mature sequences, using the flexible BLAST mapping (Altschul et al., 1990). Since the method relies completely on the presence of known homologs, the prediction accuracy will improve as more miRNAs are deposited to miRBase. However, it cannot identify species-specific miRNAs^12^.

## miRNA Validation

### Northern blot analysis

To resolve if a predicted miRNA is genuine, it is often necessary to validate it with methods other than next-generation sequencing. In this respect, Northern blot analysis can be considered as the gold standard (Lee et al., 1993; Ambros et al., 2003). First, the RNA from the cells or tissues of interest is extracted and run on a high-resolution gel. Then, the gel is treated with probes that are complementary in sequence to the predicted miRNA strand. If the strand is expressed in the cells of interest, a band corresponding to 22 nucleotides will show, and in some cases the precursor, which is around 60 nucleotides, will also show. Although this double-band constitutes compelling evidence of miRNA biogenesis, Northern blot analysis has low sensitivity, so many miRNAs that can be reliably profiled by sequencing is below Northern blot detection limit (Table 2).

**Table 2:** **Methods for miRNA validation**.

Method	Throughput	Pros	Cons
Northern blot analysis	Low	Length of transcripts observed, possibility of “double-band”	Work-intensive, lack of sensitivity
PCR-based methods	Low	Specific to transcript 3′end, sensitive	Costly for large-scale validation
Ectopic RNA hairpin expression	Low	miRNA biogenesis is directly tested	Work-intensive, impractical for large-scale validation
Association with Argonaute proteins	Low/high	Directly shows interaction with effector proteins	Method is not always specific for miRNAs
Inhibition of miRNA biogenesis pathways	Low/high	Directly shows dependence on biogenesis proteins	Knock-downs are transient and sometimes weak, generating knock-outs is time-consuming
Experimentally identified target sites	Low/high	Directly demonstrates target interaction or repression	Reporter assays are work-intensive
Conservation and population selection pressure	Sequence analysis	No wet-lab experiments required	Non-conserved miRNAs can be functional

### PCR-based methods

In contrast, real-time polymerase chain reaction (RT-PCR) methods can profile and thus validate miRNAs of very low abundance. These methods use sequence-specific primers to bind to the miRNAs and amplify them through reverse transcription and polymerase reaction (Lu et al., 2005). The abundances of amplified sequences are measured by fluorescence, and can be used to estimate the expression of the profiled miRNA. Some systems use stem-loop primers that fold around the 3′ end of the miRNA and can only amplify sequences with that particular end, increasing the specificity of the measurements (Chen et al., 2005). Although RT-PCR methods are considered reliable, the custom primers and probes for newly predicted miRNAs can be costly and the methods are rarely used to validate large sets of sequences.

### Ectopic RNA hairpin expression

In some cases, an miRNA is very lowly expressed, but researchers want to know if the miRNA biogenesis machinery would process it, were it highly expressed. It is possible to synthesize the DNA sequence of the candidate hairpin and clone it into a bacterial or viral vector (Chiang et al., 2010). The vector is then transfected into a cell culture, and the hairpin sequence is expressed. If the hairpin is recognized and cleaved by the miRNA biogenesis machinery, the predicted miRNA strand will accumulate in cells, and can then be detected by less sensitive methods, such as Northern blot analysis. A disadvantage of this method is that it is time-consuming, in that just a few miRNAs can be tested in parallel in one experiment.

### Association with Argonaute proteins

Since miRNAs associate with Argonaute proteins, showing that a predicted miRNA interacts with these proteins constitutes strong evidence of its function. There are now anti-bodies for Argonaute proteins in mammals (Ender et al., 2008), meaning that these proteins can be isolated in immuno-precipitation and their associated sRNAs studied. This profiling was previously done by Northern blot analysis or RT-PCR, but is now often done by next-generation sequencing, allowing transcriptome-wide validation. In some cases, the interaction between protein and RNA is stabilized by crosslinking (Licatalosi et al., 2008; Hafner et al., 2010), and some studies also investigate interaction with other proteins known to interact with miRNAs, such as DGCR8 (Macias et al., 2012). However, immuno-precipitation studies also have caveats as they are often performed in cell lines, which may not have the same complements of miRNAs as the tissues from which the sequences are sometimes predicted. Further, sRNAs other than miRNAs are sometimes immune-precipitated with Argonaute proteins (Ender et al., 2008), and it is not understood if these reflect genuine biological realities, or rare artifacts introduced during the experiment. Thus, the presence of an miRNA candidate in such an experiment does not constitute final evidence that it is genuine.

### Inhibition of miRNA biogenesis pathways

It is a hallmark of canonical miRNAs that they depend on the presence of Drosha, Dicer, and DGCR8 for their expression. Thus, if an miRNA candidate is depleted in cells that are void of one or more of these proteins, it constitutes strong evidence that the candidate is genuine. The expression of the proteins can be knocked down through RNA interference, where artificial sRNAs complementary in sequence to the Drosha, Dicer, or DGCR8 mRNAs are introduced into cells (Friedländer et al., 2012, 2014). The sRNAs can bind to the mRNAs and reduce protein output transiently. The genes can also be conditionally knocked out using genetic methods (Babiarz et al., 2008). In this case, Drosha, Dicer, or DGCR8 genes are deleted, leading to a collapse of the miRNA populations. Both with RNA interference and genetic methods, it is possible to use next-generation sequencing to profile miRNA expression transcriptome-wide before and after the loss of the biogenesis pathways. A limitation of the knock-down approach is that effects on the sRNA expression level are often subtle and transient (Friedländer et al., 2012). The genetic knock-outs give much clearer results, but require generation of mutant animals or cells, which is not trivial, even with the advances made with the CRISPR/Cas9 system (Cong et al., 2013; Mali et al., 2013).

### Experimentally identified target sites

Arguably, demonstrating the function of a miRNA constitutes stronger evidence than demonstrating its biogenesis or association with proteins. For this purpose, reporter constructs can be designed that are fusions of a target 3′ UTR and a reporter gene that express a marker such as luciferase (Zeng and Cullen, 2003). If the fluorescence is specifically reduced in the presence of the guide miRNA, this indicates an miRNA–target interaction. These reporter assays can be designed to simulate natural cell conditions, with endogenous miRNA and target levels and a natural number of target sites. While this method is time-consuming and only tests a single miRNA in one experiment, new genomics data can profile miRNA–target interaction transcriptome-wide (Helwak et al., 2013; Grosswendt et al., 2014). These methods use exogenous or endogenous ligases to crosslink miRNAs and their targets, and subsequently sequence these chimeric sequences, yielding information on miRNA–target pairs. These data have been found to contain novel miRNA candidates linked to mRNA sites that have typical target features (Friedländer et al., 2014).

### Conservation and population selection pressure

Some miRNAs, like *let-7*, are deeply conserved and retain almost the exact same sequence in all animals with bilateral body types, ranging from nematode to fruit fly to human (Pasquinelli et al., 2000). Thus miRNA validation is transitive: if a validated miRNA is conserved in a new species, it is likely to be genuine. There are numerous criteria for defining if an miRNA is conserved, but some parts are more likely to be under negative selection. Often homologous genome sequences from numerous species are aligned and the conservation studied to see which parts are most conserved. The nucleotides 2–8 in the 5′ end of the miRNA (the “seed”) are important for target specificity and are often conserved in evolution (Lai, 2002). In fact, miRNAs are grouping into functional gene families based on their seed sequence. The remaining part of the miRNA guide strand also confers binding specificity (Bartel, 2009) and the passenger strand is important for forming duplex with the guide. Last, the sequences flanking the two miRNA strands often exhibit some conservation, as these regions are important for the hairpin structure, and for recruiting proteins during biogenesis (Han et al., 2006). There are examples of miRNAs that are species-specific, yet have well-defined and important functions (Hu et al., 2012). In these cases, cross-species conservation patterns cannot be used, but intra-species population studies can reveal selection pressures (Friedländer et al., 2014). However, since these selection pressures can be very subtle, large numbers of novel miRNA genes are needed to detect trends, so the population approaches are not applicable to most studies. Further, sequences can to some extent be conserved by chance, so it often does not constitute definite evidence of function.

## Computational Benchmarking

Wet-lab experiments include gold standards for demonstrating that a given miRNA candidate is genuine. But computational benchmarking can give some estimates to the performance of methods to predict miRNAs, and can compare strengths and weaknesses of distinct algorithms. An advantage of benchmarking is further that it is easily undertaken by computational research groups, while performing Northern blot analyses, for instance, may require substantial investment of time and funds.

Some of the most widely used measures of prediction performance are sensitivity, specificity, and accuracy (Table 3). Sensitivity is the fraction of known distinct miRNAs in the data that are recovered by the method. Specificity is the fraction of (assumed) non-miRNA sequences that are correctly discarded by the algorithm. The false positive rate is the fraction of non-miRNA sequences that are incorrectly reported as miRNAs, or 1 – sensitivity. Accuracy is the fraction of distinct sequences that are correctly classified by the method, summing over all miRNAs and non-miRNAs. Another common measure of prediction performance is the area under curve (AUC) of receiver operating characteristic (ROC) Curve. The sensitivity is plotted as a function of the false positive rate, showing the trade-off between sensitivity and specificity. The area under the curve indicates performance, with the full area (100%) corresponding to perfect prediction, while half area (50%) corresponding to prediction that is no better than random.

**Table 3:** **Sensitivity, specificity, and accuracy**.

		miRNA state	
		Genuine miRNA	Not genuine miRNA
miRNA prediction	Positive	True positives (TP)	False positives (FP)
	Negative	False negatives (FN)	True negatives (TN)
Formulas	Sensitivity or true positive rate		TP/(TP + FN)
	Specificity or true negative rate		TN/(FP + TN)
	Accuracy		(TP + TN)/(TP + FP + FN + TN)

However, the problem of predicting miRNAs from sRNA-seq data is often a skewed one. That is, if tens of thousands of candidate hairpins are being investigated, the number of genuine miRNA precursors is typically in the hundreds. In other words, the number of negatives often vastly outnumbers the positives. Therefore, a modest reduction in sensitivity can often be tolerated, while a modest reduction in specificity can result in an unmanageable number of false positives. For instance, a reduction in sensitivity from 99 to 90% will mean a 9% loss of genuine miRNAs, while a corresponding reduction in specificity will cause a 10-fold increase in false positives, potentially rendering the resulting predictions useless. To address this, true positives and false positives are often reported as absolute numbers, to give a concrete idea of the number of sequences a user of the methods will encounter. Some methods, like miRDeep and miRDeep2, include computational controls to give the user an idea of the number of false positives generated by each run.

Most studies presenting tools to predict miRNA genes include benchmarking of their own method, often comparing it to competitor methods. A summary of these comparisons would be too comprehensive for this review; however, we have listed all the benchmarking in Table 1. However, two independent studies have been undertaken to compare the prediction performance of miRNA discovery tools. One study found miRExpress to be the most sensitive method and the mirTools suite (which uses miRDeep for prediction) to be the most accurate method (Li et al., 2012). However, we caution against relying too much on the findings of this study, as the inferred performance of the distinct tools differs widely from other performance comparisons (as referenced in Table 1). Another independent study has been undertaken to compare the prediction performance of miRDeep, miRDeep2, and miRanalyzer (updated version), which are some of the most widely used methods in the field (Williamson et al., 2013). One tool, DSAP, which quantifies miRNAs in sRNA-seq was also included in the study, but is not described here as it does not predict new miRNAs. The tools were tested against six biological datasets from cell lines and one simulated negative control data set. miRDeep2 was overall found to have the highest sensitivity, while miRanalyzer reported the most novel miRNA candidates. However, it also reported miRNAs from the simulated data, suggesting that some of the ones reported from the biological data are false positives. miRDeep had the best overall trade-off between sensitivity and specificity, as measured by AUC, followed by miRDeep2. It should be mentioned that this benchmarking just represents performance in a few use cases, and more independent studies should be undertaken to evaluate the strengths and weaknesses of the existing methods.

## Visual Inspection of Structure and Read Signature

Many tools for miRNA prediction generate graphics of the novel candidates, showing the RNA structure and the positions of the sequenced RNAs relative to the hairpin. With experience, it is possible to make estimates which of the novel candidate miRNAs can be validated in wet-lab experiments, and which will turn out to be false positive predictions. The human eye is a sensitive tool that can discriminate subtle features that are difficult to score computationally without loss of sensitivity. For instance, the miRNA hairpin structure will rarely contain large bulges, but will also rarely form a tight stem. Also, the processing of miRNA 5′ ends tends to be more precise than processing of the 3′ end (Ruby et al., 2006). Spending some time looking at gold standard known miRNAs can teach a researcher to identify these and more features. Of course, visual inspection of structure and read signature is no substitute for validation, but it can give the trained miRNA researcher an estimate of the quality of his predictions.

## Future Directions of the Field

### Resolving ambiguous sequences

Any miRNA prediction depends on read mappings that trace the sequenced RNAs to the genome loci from which they were transcribed. sRNA-seq presents difficulties that are rarely encountered in mRNA sequencing. We know from biology that each deep sequenced RNA has been transcribed from exactly one genome locus. However, when sequenced sRNAs are mapped to the reference genome, many map to more than one locus. This is in some cases because the RNA is transcribed from a gene with many copies in the genome, like a transposable element. In some cases, it will be “spurious” mappings, meaning that a short sequence can have chance matches to biologically unrelated positions in the genome, especially when the reference genome is large. A solution to the problem could be to assume that most deep sequencing reads have originated from a relatively small number of genome loci, and attempt to map the reads such that most of them locate to the fewest possible number of loci. In some concrete cases, this appears reasonable. For instance, imagine a read that maps equally well to two genome loci. One locus is a “read desert” with no other reads mapping nearby. The other locus is an rRNA gene that has thousands of reads mapping. In this case, it would seem reasonable to assume that the read should be mapped to the highly expressed rRNA locus. Some work has already been made toward overcoming these challenges. The tool SeqCluster first fuses reads that overlap in sequence in a tiled way, and subsequently maps the fused sequences to the genome (Pantano et al., 2011). These methods can resolve many, although not all, ambiguous mappings.

### Cross-mapping events

Even though next-generation sequencing quality has improved the last years, some nucleotides are inevitably called incorrectly. Similarly, sRNAs can undergo biological editing events or have untemplated nucleotides added to their 3′ ends. In these cases, an sRNA will no longer map perfectly to the genome position; it was originally transcribed from, but it may map perfectly to a distinct genome position (de Hoon et al., 2010). These wrongly mapped sRNAs will often be considered by miRNA prediction algorithms and may cause false positives. In one study, an explicit statistical model to correct these errors was developed, and numerous wrong mappings were corrected (de Hoon et al., 2010). However, this model has to our knowledge never been implemented as a user-friendly mapping tool. Ideally, such a model could be combined with a method to unambiguously trace sequenced RNAs to a single genome position (above). This would provide the sRNA community with a custom tool to handle some of the difficulties inherent in studying short sequences, and would provide an excellent platform for miRNA prediction.

### Repeat-derived miRNAs

The most commonly used tools for miRNA prediction discards mature sequences that map to many genome loci. This is a practical step to reduce the number of genome loci investigated and thus the number of false positives. However, it is well established that miRNA hairpins can arise from repetitive sequences such as transposable elements (Smalheiser and Torvik, 2005; Berezikov, 2011), and these cannot be detected by current prediction methods, unless the hairpins have diverged in sequence from the consensus repeats. Since repeat-derived sRNAs have been shown to have important functions in, for instance, the mammalian germ line (Aravin et al., 2006; Girard et al., 2006; Grivna et al., 2006; Lau et al., 2006; Watanabe et al., 2006, 2008; Tam et al., 2008), it would be interesting to investigate the prevalence and function of repeat-derived miRNAs. However, such a study could be complicated by multi-mapping problems (above) and would be much facilitated by the development of custom mapping and sequence analysis tools. Overall, the field of mapping sRNAs is understudied, and advances in this field could benefit the community.

### Reducing sRNA-seq biases

It is well established that library preparation introduces strong biases in sRNA-seq. One study has shown that artificial miRNAs introduced to a buffer in carefully controlled equal abundance give rise to numbers of reads that differ by orders of magnitude (Linsen et al., 2009). This means that some miRNAs give rise to disproportionate large numbers of reads, while others are difficult to detect and thus also more difficult to discover using sequencing. A recent study has traced these biases back to the ligase protein that joins the miRNA with sequencing adapters (Sorefan et al., 2012). miRNAs and adapters together form structures, some of which are easily ligated and some of which are difficult to ligate. In fact, since most sRNA-seq studies use the same ligase and the same adapters (from the Illumina small RNA TruSeq protocol), the miRBase database has been biased toward miRNAs that are easily ligated with this protocol. The researchers of this study has developed an alternative “high definition” protocol using pools of adapters that even out the biases, giving a more even representation of miRNAs and facilitating identification of novel sequences (Sorefan et al., 2012). As this protocol becomes more widely used in miRNAs discovery efforts, the skew in the miRBase database will, for sure, be corrected.

### Understanding the features that determine hairpin biogenesis

The human transcriptome contains more than 100,000 hairpin structures that resemble miRNA precursors (unpublished results). More than half of these are located in protein coding transcripts. Thus, while many mRNAs and miRNA primary transcripts resemble each other in being capped, poly-adenylated, and containing hairpin structures, the mRNAs are transported to the cytosol and translated, while the pri-miRNAs are cleaved into regulatory sRNAs. This mystery underlines our incomplete understanding of miRNA biogenesis: which features determine if a given hairpin is cleaved into miRNAs or left untouched? Does the presence of protein factors protect the hairpin or make it available for Drosha processing? Or does protein competition determine the hairpin fate? And which structural and sequence features of the hairpin determine which proteins are bound? Studies are unraveling these interactions (Auyeung et al., 2013) but it is clear that our understanding is still incomplete. If we would understand what hairpin features license biogenesis, we would be able to computationally predict from genome sequence, which hairpins are cleaved to miRNAs and which are left untouched.

## Conflict of Interest Statement

The authors declare that the research was conducted in the absence of any commercial or financial relationships that could be construed as a potential conflict of interest.

## Footnotes

^1^http://rth.dk/resources/dba/

^2^http://bioinfo2.ugr.es/miRanalyzer/standalone.html

^3^http://srna-workbench.cmp.uea.ac.uk/tools/analysis-tools/mircat/)

^4^https://www.mdc-berlin.de/8551903/en/research/research_teams/systems_biology_of_gene_regulatory_elements/projects/miRDeep

^5^http://www.australianprostatecentre.org/research/software/mirdeep-star

^6^http://www.lgm.upmc.fr/mirena/index.html

^7^http://omictools.com/mirevo-s962.html

^8^http://mirexpress.mbc.nctu.edu.tw

^9^http://flybuzz.berkeley.edu/miRTRAP.html

^10^http://www.ncrnalab.dk/#mirdentify/mirdentify.php

^11^http://www.uea.ac.uk/computing/mirplex

^12^https://bioinformatics.mpi-bn.mpg.de
